# Continuity of primary care and end-of-life care costs in dementia: a retrospective cohort study

**DOI:** 10.3399/BJGP.2025.0218

**Published:** 2026-01-13

**Authors:** Javiera Leniz, Peter May, Martin Gulliford, Katherine E Sleeman

**Affiliations:** 1 School of Public Health, Pontificia Universidad Católica de Chile, Santiago, Chile; 2 School of Medicine, Trinity College Dublin, Dublin, Ireland; 3 Cicely Saunders Institute of Palliative Care, Policy and Rehabilitation, King's College London, London, UK; 4 School of Life Course and Population Sciences, King's College London, London, UK

**Keywords:** continuity of care, dementia, end-of-life care, general practice, healthcare costs

## Abstract

**Background:**

End-of-life dementia care costs are expected to rise as populations age. Higher continuity of care with GPs is associated with reduced hospital admissions at the end of life, but the impact on costs is not known.

**Aim:**

To explore the association of continuity of primary care on hospital and general practice costs in the last year of life among people with dementia.

**Design and setting:**

A retrospective cohort study using a primary care dataset linked with national hospital and mortality records. Adults (aged >18 years) who died in England between 2009 and 2018 with a diagnosis of dementia were included.

**Method:**

The Continuity of Care Index (COCI) of GP contacts in the last year of life was calculated, which measures patterns of care across GPs. Hospital and general practice costs were calculated using average national tariffs. Costs were modelled using a multivariable generalised linear model, estimating the average marginal effect of perfect continuity over non-continuity of care.

**Results:**

In total, 32 799 people were included. The mean age at death was 86.60 years (standard deviation [SD] 8.04 years), 64.2% (*n* = 21 057) were female, and 56.6% (*n* = 18 556) lived in care homes before death. The average COCI score was 0.38 (SD 0.25). People with perfect continuity had on average £2097 (95% confidence interval = 1319 to 2875) lower total costs in the last year than those with non-continuity of care.

**Conclusion:**

Continuity of care with GPs is associated with lower total costs and might contribute to reduce hospital admissions and costs among people with dementia in their last year of life.

## How this fits in

Continuity of care with the GP has been associated with lower hospital admissions and costs among older adults and people with dementia, but the impact on end-of-life care costs was unknown. As end-of-life care and hospital costs increase rapidly with proximity to death, it is important to understand to what extent continuity of care in primary care may have an effect on end-of-life care costs. The results of the current study show that people with better levels of continuity of care with GPs have lower total costs in the last 12 months of life than those with worse levels of continuity of care. Prioritising having appointments with the same GP among people with dementia approaching the end of life could potentially reduce unnecessary admissions to hospital and costs in the last year of life.

## Introduction

Dementia is a major public health concern, affecting >60 million people worldwide and posing significant challenges to healthcare systems.^1^ The disease trajectory is characterised by slow and progressive cognitive and functional decline,^2^ which is associated with an increase in symptoms and healthcare needs.^3–6^ Towards the end of life, people with dementia experience a large increase in emergency department visits and hospital admissions,^7–9^ and higher healthcare costs than older adults without dementia.^10–12^ End-of-life hospital admissions have been associated with poorer outcomes in people with dementia,^13,14^ and reducing reliance on acute hospital care through shifting the balance of care into the community is a national policy priority.^15^

Continuity of care can be understood as a continuous caring relationship between a patient and a physician.^16,17^ A broader definition of continuity of care includes the level of coordination of care across providers.^16^ Continuity of care indices can be calculated from administrative data that captures the extent to which a patient has most of their encounters with the same physician.^18^ Higher continuity of care scores have been associated with lower emergency department visits, lower rates of hospital admission in people with dementia and older adults,^19–24^ and lower hospital costs, although this has not been investigated in the last year of life specifically.^19,21,25,26^

A previous study found that continuity of care with GPs was associated with a lower risk of having multiple hospital admissions in the last year of life among people who died with dementia in England.^27^ The impact of continuity of primary care on costs in the last year of life is not known, but since costs — in particular hospital costs — increase rapidly with proximity to death,^28^ there is potential for continuity of care to both improve patient outcomes and reduce system expenditures.

The aim of this study was to explore the association of continuity of primary care on hospital and general practice costs in the last year of life among people with dementia in England.

## Method

### Design and data sources

This was a nationwide population-based retrospective cohort study in England using the Clinical Practice Research Datalink (CPRD), linked with hospital records (Hospital Episode Statistics [HES]) and mortality data from the Office for National Statistics (ONS).

### Population

This study included adults (aged >18 years) who died between 1 January 2009 and 31 December 2018, had a dementia diagnosis, and a 12-month before-death registration period in a general practice with continuous high-quality data based on CPRD quality checks.^29^ Dementia diagnosis was identified either from primary care records (using Read codes)^30^ or hospital records (using International Statistical Classification of Diseases and Related Health Problems 10th Revision [ICD-10] codes), based on previous studies (see Supplementary Box S1).^31,32^

### Outcome

The outcome was hospital and general practice costs of care in the last year of life from the NHS perspective. For hospital costs, Healthcare Resource Groups 4 (HRG4) codes available in the HES dataset for each hospital admission that occurred in the last year of life were used. HRG4 codes are derived from the main diagnosis, procedures, age, and severity of the condition, and categorise similar clinical treatments that should cost an equivalent amount to deliver.^33^ Each HRG4 code is associated with a national tariff, which is updated annually based on average costs of providing that service to NHS patients in England. When a valid HRG4 code was not available for an admission (5657 out of 61 351 admissions), the median unit cost of all admissions by year, main diagnosis, age group, sex, Index of Multiple Deprivation (IMD) quintile, and region in the sample was used.

For general practice costs, the cost of each consultation in the practice was calculated using costs reported by the Personal Social Services Research Unit (PSSRU).^34^ This was based on the estimated cost per surgery consultation lasting 10 minutes in 2024 for face-to-face GP consultations, and the average GP telephone call consultation for telephone consultations. For nurse consultations at the general practice, the average face-to-face cost per hour for qualified nurses, as reported by the PSSRU,^34^ assuming 30 minutes length per consultation, was used. Home visits and out-of-office consultations were not included.

Costs were reported in pound sterling (£) and inflated to 2024 costs.

### Continuity of care

The COCI score with GPs was calculated, as personal anonymised codes were only available for GPs and not for other healthcare professionals in the practice. The COCI score measures the extent to which consultations during a certain period of time are concentrated with the same physicians, taking into account how different patterns of consultations provide different levels of continuity of care. It has a range from 0 to 1 (1 meaning better levels of continuity of care).^35^ The COCI score was calculated only for consultations with GPs (face-to-face or telephone consultations) during the last year of life. As the COCI score cannot be calculated with <2 consultations, participants (*n* = 915) with <2 consultations with a GP during the last year of life were excluded. Therefore, people who had consultations with nurses but only one or no consultations with a GP were excluded.

### Covariables

Factors associated with healthcare use at the end of life were examined based on previous research and theoretical models.^27,36,37^ The age at death using the year of death and year of birth was calculated. Sex, general practice region, and 2011 England and Wales rural–urban classification were extracted from CPRD. An IMD quintile for each participant was defined based on the lower layer super output areas from the latest available participants’ postcode of residence.

The underlying cause of death and date of death were identified from ONS. The underlying cause of death was grouped into ICD-10 block codes (see Supplementary Table S1). The number of comorbidities (excluding dementia) were calculated using the count of chronic diseases from the Quality and Outcomes Framework Read codes rules.^30,38^ Read codes were used to identify whether participants had a record of living in a care home (nursing or residential care home), based on a previous publication (see Supplementary Table S2).^39^ People were identified with a code indicating the participant was included in the palliative care register at any point before the last 90 days of life, to recognise people who had been identified by their GPs as having palliative care need, as in previous publications.^27,40,41^

### Analysis

For the descriptive analysis, the COCI score was categorised in low (0–0.126), medium (0.127–0.632), and high (0.633–1) levels of continuity of care based on the mean plus or minus 1 standard deviation (SD) to facilitate interpretation. The mean SD and frequencies were calculated by level of continuity of care and the whole sample for each continuous and categorical variable in the analysis, respectively.

Hospital (elective and non-elective) and general practice costs in the past 3 and 12 months before death were calculated. Total costs were calculated by adding total hospital and GP practice costs.

For the primary analysis, the effect of the COCI score as a continuous variable on hospital, general practice, and total costs during the last 3 and 12 months before death was evaluated. Costs were modelled using a multivariable generalised linear model (GLM) with a gamma distribution and a log link, with data clustered by general practices. As hospital costs were zero inflated (high proportion of people with no admissions and therefore zero hospital costs), a two-part model was employed: a logistic regression for zero costs and a GLM for non-zero hospital costs. All models were adjusted for age, sex, IMD quintile, region, year of death, rurality, number of comorbidities, residence in a care home during the last year of life, number of consultations with the GP in the last year of life, and identification of palliative care needs before the last 90 days of life, based on previous research.^27^ The average marginal effect of continuity of care and the predicted average costs at each decile of the COCI score were estimated, holding other covariates at their means. The marginal effect can be interpreted as the estimated difference in the cost when increasing the level of continuity of care from 0 (all contacts the patient has in the last year of life were with a different GP) to 1 (all contacts with the same GP). To calculate 95% confidence intervals (CIs) for the average marginal effect and predicted average costs, 100 bootstrap samples were used. As the COCI score has been shown to be less stable when participants have <4 contacts, the same final model excluding those participants was performed as a sensitivity analysis.

**Figure 1.**
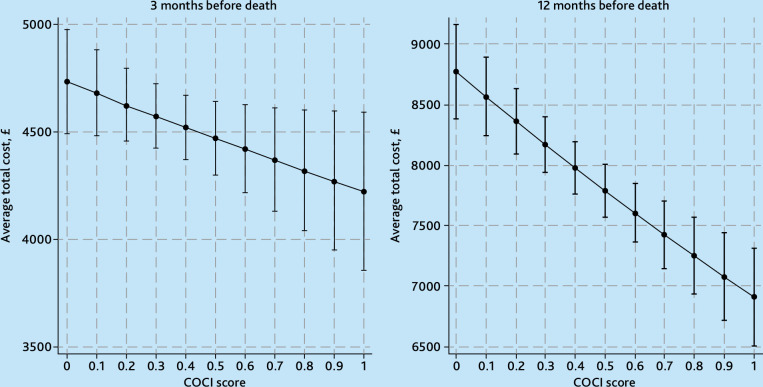
Average estimated total costs per person (hospital plus general practice costs) in the last a) 3 and b) 12 months before death, by decile of COCI in the last year of life. Adjusted by age, sex, Index of Multiple Deprivation quintile, region, year of death, rurality, number of comorbidities, residence in a care home during the last year of life, number of consultations with the GP in the last year of life, and identification of palliative care needs before the last 90 days of life. COCI = Continuity of Care Index.

## Results

In total, 33 714 people with dementia who died between 2009 and 2018 and were registered in a general practice during the last year of life were identified in CPRD records. After excluding 915 participants with <2 consultations in the GP practice during the last year of life, 32 799 people were included in the analysis. Of these, 8817 (26.9%) did not have any hospital admissions in the last year of life (see Supplementary Figure S1).

### Characteristics of the cohort

Of the 32 799 participants, the mean age at death of the cohort was 86.60 (SD 8.04) years, 64.2% (*n* = 21 057) were female, 21.7% (*n* = 7122) lived in an area from the least deprived quintile, and 56.6% (*n* = 18 556) lived in a care home before death. The most common underlying cause of death was dementia (36.9%, *n* = 12 107) followed by cerebrovascular and ischaemic heart diseases, and only 9.6% (*n* = 3135) of participants were recognised as having palliative care needs at least 90 days before death (Table 1).

**Table 1.** Sample sociodemographic and clinical characteristics by level of continuity of care with the GP^a^

		Continuity of Care Index score, *n* (%)^b^		
**Characteristic**	**All sample**	**Low**	**Medium**	**High**
**Total**	32 799	3847	23 556	5396
**Age, mean (SD)**	86.60 (8.04)	86.93 (8.17)	86.57 (8.01)	86.45 (8.08)
**Age, years**				
≤65	625 (1.9)	75 (2.0)	446 (1.9)	104 (1.9)
66–75	1939 (5.9)	210 (5.5)	1385 (5.9)	344 (6.4)
76–85	10 151 (31.0)	1152 (30.0)	7312 (31.0)	1687 (31.3)
86–95	16 764 (51.1)	1953 (50.8)	12 093 (51.3)	2718 (50.4)
≥96	3320 (10.1)	457 (11.9)	2320 (9.9)	543 (10.1)
**Sex**				
Male	11 742 (35.8)	1277 (33.2)	8491 (36.1)	1974 (36.6)
Female	21 057 (64.2)	2570 (66.8)	15 065 (64.0)	3422 (63.4)
**IMD quintiles**				
1 (least deprived)	7122 (21.7)	788 (20.5)	5219 (22.2)	1115 (20.7)
2	7276 (22.2)	745 (19.4)	5166 (21.9)	1365 (25.3)
3	7658 (23.4)	877 (22.8)	5421 (23.0)	1360 (25.2)
4	5720 (17.4)	738 (19.2)	4056 (17.2)	926 (17.2)
5 (most deprived)	5012 (15.3)	698 (18.1)	3688 (15.7)	626 (11.6)
Missing	11 (0.03)	1 (0.03)	6 (0.03)	4 (0.07)
**Geography**				
Urban	28 100 (85.7)	3393 (88.2)	20 284 (86.1)	4423 (82.0)
Rural	4699 (14.3)	454 (11.8)	3272 (13.9)	973 (18.0)
**Underlying cause of death**				
Dementia	12 107 (36.9)	1441 (37.5)	8704 (37.0)	1962 (36.4)
Cancer	2872 (8.8)	273 (7.1)	2137 (9.1)	462 (8.6)
Cerebrovascular disease	3497 (10.7)	398 (10.4)	2527 (10.7)	572 (10.6)
Ischaemic heart disease	2422 (7.4)	294 (7.6)	1698 (7.2)	430 (8.0)
Influenza and pneumonia	1747 (5.3)	197 (5.1)	1258 (5.3)	292 (5.4)
Other	10 150 (31.0)	1244 (32.3)	7228 (30.7)	1678 (31.1)
Missing	4 (0.01)	0 (0.0)	4 (0.02)	0 (0.0)
**Comorbidities, mean (SD)**	2.25 (1.60)	2.18 (1.62)	2.30 (1.61)	2.08 (1.54)
**Lived in a care home**				
No	14 243 (43.4)	1670 (43.4)	10 118 (43.0)	2455 (45.5)
Yes	18 556 (56.6)	2177 (56.6)	13 438 (57.1)	2941 (54.5)
**Identification of palliative care needs before last 90 days of life**				
No	29 664 (90.4)	3540 (92.0)	21 297 (90.4)	4827 (89.5)
Yes	3135 (9.6)	307 (8.0)	2259 (9.6)	569 (10.5)
**Continuity of Care Index score, mean (SD)**	0.38 (0.25)	0.07 (0.05)	0.33 (0.14)	0.83 (0.13)
**Contacts with GPs at the general practice in the last year of life, mean (SD)**	16.57 (11.49)	12.77 (10.65)	17.59 (11.60)	14.80 (10.79)

^a^Year of death and region distribution for the entire cohort and by continuity of care level are available in Supplementary Table S3. ^b^Unless otherwise specified. IMD = Index of Multiple Deprivation. SD = standard deviation.

The average COCI score in the entire cohort was 0.38 (SD 0.25). The age and sex distribution across COCI score categories was similar, but people with lower COCI scores (0–0.126) were more likely to live in more deprived and urban areas, and less likely to have been recognised as having palliative care needs than those with higher COCI scores (0.633–1) (Table 1). COCI scores varied by region and year of death (see Supplementary Table S3).

### Healthcare use and costs at the end of life

People in the sample had an average of 0.11 (SD 0.87) elective and 0.85 (SD 0.91) non-elective admissions in the last 3 months of life, with a mean length of stay per admission of 1.23 (SD 17.95) and 7.94 (SD 14.38) days, respectively. Average non-adjusted costs per person in the last 3 months of life were £547 (SD 8712) for elective admissions and £4000 (SD 9392) for non-elective admissions. The average number of total contacts with the general practice was 7.87 (SD 6.00), and total average costs per person were £341 (SD 259) in the last 3 months of life (see Supplementary Table S4).

In the last 12 months of life, the average number of elective and non-elective admissions rose to 0.37 (SD 3.74) and 1.50 (SD 1.58), with a mean length of stay per admission of 1.88 (SD 9.94) and 9.19 (SD 13.96) days, respectively. Average non-adjusted costs per person in the last 12 months of life were £1109 (SD 6475) for elective admissions and £6664 (SD 9031) for non-elective admissions. People had an average number of contacts with the general practice of 21.62 (SD 14.99) in the last 12 months of life, and total average costs per person were £924 (SD 636) (see Supplementary Table S4).

### The effect of continuity of care on costs

The level of continuity of care with the GP in the last year of life was significantly associated with lower total costs in the last 12 months but not in the last 3 months of life. Figure 1 shows the average estimated total costs of care in the last 3 months and 12 months before death by decile of COCI score. The average total costs per person in the last year of life were estimated at £8775 (95% CI = 8386 to 9164) for people who had the worst level of continuity of care (score of 0) versus £6913 (95% CI = 6510 to 7315) for those who had the best level of continuity of care (score of 1). A similar pattern was observed for costs in the last 3 months of life, although the difference was smaller and not statistically significant (Figure 1 and Supplementary Table S5).

On average, total costs in the last year of life for people with a COCI score of 1 were estimated to be £2097 lower than for people with a COCI score of 0. In other words, increasing the COCI score from 0 to 1 could result in an average total cost reduction of £2097 (95% CI = 1319 to 2875) per person in the last 12 months of life. Total costs in the last 3 months of life for people with a COCI score of 1 were on average £564 lower than for people with a COCI score of 0, but the 95% CI indicates that the difference in cost between a COCI of 0 and 1 could range between a £1155 reduction and an £28 increase (Table 2).

**Table 2.** Effect of the COCI score on hospital, general practice, and total costs per person in the last 3 and 12 months before death, after adjusting by confounding factors

Characteristic	Adjusted model			Marginal effect,^a^ £	
	Coefficient^b^	95% **CI^c^**	*P*-value	Margins	95% **CI**
**3 months before death**					
Hospital costs	1.021	0.930 to 1.121	0.661	–418	–918 to 83
General practice costs	0.922	0.875 to 0.971	0.002	–30	–49 to –11
Total costs (Hospital and General Practice)	0.892	0.791 to 1.006	0.062	–564	–1155 to 28
**12 months before death**					
Hospital costs	0.884	0.820 to 0.952	0.001	–1764	–2452 to –1076
General practice costs	0.946	0.899 to 0.996	0.034	–57	–110 to –4
Total costs (Hospital and General Practice)	0.788	0.722 to 0.859	<0.001	–2097	–2875 to –1319

a Marginal effect can be interpreted as the estimated difference in the cost when increasing the COCI score from 0 (worst) to 1 (best). ^b^The exponentiated coefficient estimated from the GLM with a gamma distribution and a log link. The log link function in the GLM models the logarithm of the expected cost as a linear function of the predictors. The exponentiated coefficient represents the rate between the mean costs for COCI score = 1 over COCI score = 0. In other words, for people with a COCI score of 1 (best level of continuity of care), the expected total costs in the 12 months before death would be 21.2% (1 – 0.788) lower, holding all other variables constant. ^c^The 95% CI represents the 95% exponentiated CIs of the coefficient estimated from the GLM. CIs were estimated from 100 bootstrap samples. All values are adjusted by age, sex, Index of Multiple Deprivation quintile, region, year of death, rurality, number of comorbidities, residence in a care home during the last year of life, number of consultations with the GP in the last year of life, and identification of palliative care needs before the last 90 days of life. COCI = Continuity of Care Index. GLM = generalised linear model.

The level of continuity of care with the GP was associated with lower general practice costs both in the last 3 months and in the last 12 months before death; however, it was only significantly associated with lower hospital costs in the last 12 months (Table 2 and Supplementary Table S6). The sensitivity analysis performed among people with ≥4 contacts with the GP in the last year of life showed similar results (see Supplementary Table S7).

## Discussion

### Summary

This large population-based cohort of people who died with dementia in England found that people with higher continuity of care with the GP had lower average total costs (hospital and general practice costs) in the last 12 months before death. These results suggest that increasing the level of continuity of care from a score of 0 (all contacts in the last year of life with a different GP) to a score of 1 (all contacts with the same GP), could result in an average total cost reduction between £1319 and £2875 per person in the last 12 months of life.

### Strengths and limitations

To the authors’ knowledge, this is the first study to explore the effect of continuity of care on end-of-life hospital and general practice costs in England. An important strength is the use of a large nationwide population-based cohort linked with hospital and death certificate records. Nevertheless, this study has some limitations. The study considered costs from a healthcare system perspective and only had access to inpatient hospital and general practice costs. Although inpatient hospital costs represent an important component of end-of-life care costs, not including informal or residential care costs is likely to underestimate total end-of-life care costs from a societal perspective.^42^

The authors of the current study cannot rule out the possibility of residual confounding, as people with good continuity of care may also have higher self-efficacy and strongest proclivity to avoid hospital care. Other aspects might influence continuity of care at the end of life that are not measured in this study, such as access to social support, severity of diseases, and poor symptoms control.

The COCI score used in the current study has some limitations: it does not capture the nature of the doctor–patient relationship, as it does not address the quality of the encounter or relationship. A patient might have a pattern of consultations that represents a good level of continuity, but with the patient’s less-preferred physician. The COCI score is sensitive to the total number of visits, as people with more visits will tend to have lower COCI scores.^22^ The Index also does not address coordination of care across providers, an important aspect of continuity of care from a broader perspective.^16^ Nevertheless, these are common limitations among continuity of care measures from administrative data.^35^

### Comparison with existing literature

Four other studies have explored the relationship between continuity of care and healthcare costs in older adults or people with dementia, although none investigated the last year of life specifically. Three of them were based in the US^19,21,43^ and one in Taiwan.^25^ Chao *et al* found that higher levels of continuity of care were significantly associated with lower outpatients and hospital costs in people with dementia from Taiwan.^25^ Lei *et al* estimated that a 0.1 higher COCI score could reduce inpatient costs between $2506 and $688 and reduce emergency department costs between $174 and $64 among people with dementia.^43^ The current study’s findings are consistent with results from these other studies in a dementia population in different countries and stages of the disease.

The literature suggests continuity of care might contribute to reduce hospital admissions and costs by improving communication, the identification of patient’s needs, and taking a more person-centred approach,^44–46^ all important aspects for dementia end-of-life care. People with dementia experience complex, demanding, and fluctuating care needs when approaching the end of life that are difficult to address.^47–49^ Families and patients with dementia frequently express their preferences for receiving care in the community but have to go to emergency rooms because of the lack of support from a healthcare provider that ‘knows them well’ during a crisis.^49–51^ Better continuity of care with the GP might contribute to address that need.

The current study’s results show that people with dementia with better levels of continuity of care had lower hospital costs than those with worse levels of continuity of care, mainly explained by lower numbers of non-elective hospital admissions. This association was significant for the last 12 months of life but not for the last 3 months. Healthcare professionals recognise that knowing the person with dementia facilitates interpretation of non-verbal communication, which helps to better address symptoms and needs.^52^ This might contribute to avoiding unnecessary admissions to hospital. However, emergency admissions closer to death might represent events that are not possible to manage at home and therefore not easy to prevent.

### Implications for research and practice

This study’s findings suggest that improving the level of continuity of primary care could contribute to reduce unnecessary hospital admissions and hospital costs among people with dementia at the end of life. However, continuity of care has been declining in England. The proportion of patients reporting seeing their preferred GP regularly declined from 56.7% to 47.3% between 2012 and 2017.^53^ General practice redesign and the increasing workload have been suggested to have a negative impact on continuity of care, potentially explaining these findings.^54^ Although continuity of care might have a negative impact on access,^55,56^ informational and management continuity, or prioritising continuity of care for those who might benefit the most, might contribute to increasing continuity without compromising access.^56^ Recently, NHS England has introduced financial incentives to general practices that stratify and identify patients that would benefit the most from continuity of care.^57^ General practices should consider people approaching the end of life and people with dementia among their priority groups. However, it is unclear to what extent these policy measures could have an effect on the timeliness of care and therefore should be carefully monitored.

Although the study did not address the cost of increasing the level of continuity of care, these findings suggest this might not be significant, as higher continuity of care was associated with lower general practice costs. Previous research suggests that other healthcare professionals such as community and Admiral nurses play an important role in dementia care.^58,59^ It is likely that a combination of continuity with the GP and other healthcare professionals in the community has a positive impact on quality of care and can contribute to reducing hospital admissions.

In conclusion, people with dementia who have higher levels of continuity of care with their GP have significantly lower inpatient and GP costs during the last year of life. The findings suggest that improving the level of continuity of care in primary care might contribute to reduce hospital admissions and costs in the last year of life.

## Data

The data that support the findings of this study are available from CPRD. Access to CPRD data is subject to protocol approval by an Independent Scientific Advisory Committee (ISAC). The protocol of this study was approved by the ISAC in April 2021 (protocol number: 20_031).
